# Evidence and rationale for the World Health Organization recommended standards for Japanese encephalitis surveillance

**DOI:** 10.1186/1471-2334-9-214

**Published:** 2009-12-29

**Authors:** Susan Hills, Alya Dabbagh, Julie Jacobson, Anthony Marfin, David Featherstone, Joachim Hombach, Pem Namgyal, Manju Rani, Tom Solomon

**Affiliations:** 1 PATH, Seattle, WA, USA; 2 World Health Organization (WHO), Geneva, Switzerland; 3 Centers for Disease Control and Prevention, Fort Collins, CO, USA; 4 WHO Regional Office for South-East Asia, New Delhi, India; 5 WHO Regional Office for Western Pacific, Manila, Philippines; 6 University of Liverpool, Liverpool, UK; 7 Current address: Centers for Disease Control and Prevention, Fort Collins, CO, USA; 8 Current address: Bill and Melinda Gates Foundation, Seattle, WA, USA; 9 Current address: Washington State Department of Health, WA, USA

## Abstract

**Background:**

Japanese encephalitis (JE) is the most important form of viral encephalitis in Asia. Surveillance for the disease in many countries has been limited. To improve collection of accurate surveillance data in order to increase understanding of the full impact of JE and monitor control programs, World Health Organization (WHO) Recommended Standards for JE Surveillance have been developed. To aid acceptance of the Standards, we describe the process of development, provide the supporting evidence, and explain the rationale for the recommendations made in the document.

**Methods:**

A JE Core Working Group was formed in 2002 and worked on development of JE surveillance standards. A series of questions on specific topics was initially developed. A literature review was undertaken and the findings were discussed and documented. The group then prepared a draft document, with emphasis placed on the feasibility of implementation in Asian countries. A field test version of the Standards was published by WHO in January 2006. Feedback was then sought from countries that piloted the Standards and from public health professionals in forums and individual meetings to modify the Standards accordingly.

**Results:**

After revisions, a final version of the JE surveillance standards was published in August 2008. The supporting information is presented here together with explanations of the rationale and levels of evidence for specific recommendations.

**Conclusion:**

Provision of the supporting evidence and rationale should help to facilitate successful implementation of the JE surveillance standards in JE-endemic countries which will in turn enable better understanding of disease burden and the impact of control programs.

## Background

Japanese encephalitis (JE) is a severe disease, with most of the South-East Asia and Western Pacific WHO-defined geographical regions at risk. Although JE is acknowledged as a major public health problem, understanding of the full impact of the disease is limited by lack of accurate and comprehensive disease burden data. It has been estimated that about 175,000 JE cases would occur annually in children under 15 years of age in the absence of immunization [1]. Reported JE case numbers are much lower, in part due to JE vaccination programs in several large countries including China, Japan and Viet Nam. However, with many JE-endemic countries not conducting surveillance or not reporting case numbers to the WHO, disease burden in many countries is not well understood and an accurate estimate of the current global JE case load is not available. Not only are data lacking, but surveillance data gathered and reported are not standardized. Reporting may be based on laboratory-confirmed cases, viral encephalitis cases, acute encephalitis cases occurring during a recognized JE season, or on other mixed criteria. This lack of consistency can make interpretation of data difficult.

The WHO publishes standards for surveillance for selected vaccine-preventable diseases [2]. Surveillance standards provide guidance for implementation or strengthening of surveillance systems and encourage consistency in data collection and reporting, thus allowing for comparability of the information gathered. In 2002 following an international JE meeting held in Thailand, a group of organizations formed a JE Core Working Group (CWG) to strengthen and facilitate co-ordination of JE prevention and control activities. One of the group's initial activities was drafting JE surveillance standards, and the final version was ultimately published in August 2008 [2]. The CWG thought it was important to document the process of developing the standards and the underlying rationale for the recommendations made. Surveillance standards developed previously have not always documented such information, and misunderstandings or controversies have sometimes resulted [3-5]. The objective of this paper is to provide the rationale for, and describe the evidence that supports, the recommendations made in the Standards. Presentation of the rationale will also allow the future update and interpretation of these standards as additional data become available.

## Methods

### Process of development of the Standards

In April 2003, at a meeting of the JE CWG at the WHO headquarters, discussions began on the JE surveillance standards. WHO surveillance standards for vaccine-preventable diseases follow a standard format, including recommendations on case definition (clinical and laboratory criteria), types of surveillance, data to be collected and recommended surveillance performance indicators. The CWG initially defined a series of important questions to be addressed within each topic area (Table 1). A systematic search was undertaken for literature to address these questions, which was reviewed at meetings at WHO headquarters in July 2004, in Thailand in April 2005, and in smaller group teleconferences. Emphasis was placed on the feasibility of implementation of the Standards in developing country settings, and different surveillance strategies were included to ensure relevance to countries based on their local surveillance and laboratory infrastructure, and JE control status [6].

**Table 1:** Topics and issues considered in development of the Japanese encephalitis (JE) surveillance standards

TOPIC	ISSUES CONSIDERED
**Clinical case definition**	Is it possible to clinically differentiate JE from other causes of acute encephalitis? What symptoms and signs should be included in the case definition for acute encephalitis syndrome (AES)? Should parameters such as age and seasonality be included in the case definition?

**Laboratory criteria for confirmation**	What samples should be used for confirming JE infection, and which are preferred? What tests are appropriate for confirming JE infection? What timing should be recommended for collection of samples? What factors need to be considered in testing for JE virus infection and in interpreting test results?

**Case classification**	What case classifications are appropriate based on clinical, epidemiological and laboratory findings?

**Types of surveillance**	What is the best model for JE surveillance and are different types of surveillance appropriate in different settings?

**Minimum data elements, analyses and reporting**	What data should be collected for analysis and reporting for routine surveillance purposes? What targets should be used to monitor the quality of JE surveillance?

**Special aspects**	What other special aspects of clinical and laboratory surveillance for AES and JE should be considered?

In January 2006 a field test version of the Standards was published electronically. During 2006, feedback was sought from surveillance and disease control managers, Expanded Program on Immunization (EPI) managers, and field staff. Several countries including Indonesia, Viet Nam, and India, piloted the standards, either broadly or in defined geographical areas. Other forums and meetings were also used for consultation, including a WHO South-East Asia regional JE workshop in India in April 2006. All information gathered was reviewed at a CWG meeting in Viet Nam in April 2007 and amendments to the Standards were proposed. A concluding review was undertaken by the WHO, entailing review by independent external experts, before final publication in August 2008.

### Intended users of the Standards

The Standards are primarily intended for use by staff responsible for the surveillance of vaccine-preventable or communicable diseases. However the guidelines also have implications for clinicians, laboratory managers, technicians, EPI staff, and other public health professionals. They are principally for surveillance purposes; the CWG recognized that the clinical and diagnostic criteria for JE for individual patient management may be more rigorous and vary in different settings.

### Type of evidence for the recommendations

A simple, hierarchical grading system has been used in this paper to categorize the level of evidence supporting recommendations in the JE surveillance standards (Table 2). It is based on a published system used previously by a WHO-convened group [7]. Although the recommendations do not relate to clinical interventions, services or policies, we felt that providing a simple indicator of evidence would be helpful for users of the Standards.

**Table 2:** Levels of evidence for recommendations in the surveillance standards

**Category I**	Strongly recommended and strongly supported by well-designed experimental or epidemiological studies.
**Category II**	Recommended on the basis of theoretical rationale and suggestive, descriptive evidence.
**Category III**	Recommended on the basis of expert consensus and theoretical rationale.

## Results

The evidence gathered to help develop the surveillance standards, including the findings from the literature reviews, and rationale for the recommendations, are presented below. Each component of the standards for which recommendations were made is discussed, including the case definition for surveillance, types of surveillance, data to be collected and surveillance performance indicators. The level of evidence for each recommendation or topic is noted.

### Clinical case definition

Is it possible to differentiate JE from other causes of acute encephalitis clinically?

The most commonly recognized presentation of JE virus (JEV) infection is acute encephalitis [8-11], and it is usually clinically indistinguishable from other causes of acute encephalitis syndrome (AES) [12-15]. The Standards therefore recommend clinical syndromic surveillance to detect patients with AES, and subsequent laboratory confirmation of JEV infection. *[Category II]*

What symptoms and signs should be included in the case definition for AES?

The usual clinical presentation of acute encephalitis is fever, associated with nausea and vomiting, and neurologic symptoms and signs including headache, lowered level of consciousness, seizures and/or focal neurological signs [16,17]. Case definitions used for acute encephalitis in research studies vary, but often focus on the presence of at least two specific features, including fever (often an essential component), altered consciousness, headache, seizures, focal neurological signs, neck stiffness or typical laboratory parameters [18-20]. Based on this information, and recognizing the need for a simple case definition to facilitate surveillance activities, the clinical AES definition is defined as fever and one or both of a change in mental status or new onset of seizures. *[Category II] *Simple febrile seizures are excluded.

Subsequent evaluation of this definition using a cohort of 63 Vietnamese patients with laboratory-confirmed JE established its sensitivity and specificity as 65% and 39%, respectively [15]. Acute flaccid paralysis (AFP) and meningism are also common with JEV infection [10,12,19,21-23]. Adding either of these criteria improved the sensitivity to over 85%, but decreased specificity to 26% or 23% with AFP and meningism, respectively. The CWG did not consider it justifiable to change the case definition to include AFP and meningism, as the loss of specificity would result in substantially greater costs and efforts for surveillance due to laboratory testing of the additional cases.

Should parameters such as age and seasonality be included in the case definition?

JE is most commonly seen in Asia in children up to 15 years of age. A high percentage of persons have acquired immunity by this age due to ongoing environmental JEV transmission [1,24-27]. However, cases can occur in non-immune adults and are particularly apparent in this age group when the virus enters new areas [28-30]. In addition, age distribution may shift to higher ages when childhood JE immunization programs have been implemented [31-33]. The case definition therefore describes a case as one occurring in a "person of any age". *[Category I] *However in some countries, particularly those at an early stage of JE control, it may be more feasible and cost-effective to target surveillance to the under-15 year age group or the group classified as 'paediatric'.

JEV transmission may be seasonal or year-round [12,32,34-38]. As AES is caused by multiple different pathogens, AES cases occur throughout the year. To avoid lack of reporting of cases at certain times of the year, particularly during periods considered to be outside the JE season, the case definition refers to cases occurring "at any time of the year". *[Category I]*

Based on the considerations above, a final AES case definition was derived (Additional file 1).

### Laboratory criteria for confirmation

Patients meeting the clinical case definition of AES should have laboratory testing conducted to determine if the cause of illness is JEV or another agent. Sample collection and testing must be as complete as possible.

What samples should be used for confirming JE infection, and which are preferred?

Detection of immunoglobulin M antibody (IgM) in cerebrospinal fluid (CSF) is considered the most reliable method for JE diagnosis [39]. The presence of JEV IgM in CSF indicates infection of the CNS, proving JEV is the cause of the patient's encephalitic illness. If CSF is not available, the diagnosis can be confirmed by presence of JEV IgM in serum. JEV IgM in serum indicates the patient is infected with JEV. It does not confirm the patient's encephalitis is caused by JEV--there is a possibility the patient could have simultaneous asymptomatic JEV infection and AES due to another cause. If diagnosis is by serum alone there is also a greater risk of a falsely positive test result due to cross-reactivity with co-circulating flaviviruses. However, studies have shown most patients who present with AES during the JE season and have JEV IgM in serum also have it in CSF and do not have evidence for a different CNS infection [18,19,40].

The CWG gave careful consideration to whether a positive CSF result should be the only criteria used to define a "laboratory-confirmed" case in the Standards. However, if only CSF results were considered, surveillance would lack sensitivity, with one third of JE cases or more possibly missed because of both diagnostic testing and sample collection issues as described below.

Firstly, JEV IgM may not be detectable in the CSF of all patients at presentation to hospital. Studies indicate up to about 30% of patients may not have detectable JEV IgM on admission or within a few days of symptom onset [39-44]. Although JEV IgM continues to increase and is usually detectable in CSF by day 7 to 8 of illness, a second (convalescent) CSF specimen is not, in general, indicated for clinical management purposes and cannot be recommended for surveillance purposes alone, so many cases would be missed.

Secondly, lumbar puncture (LP) is not always possible because of medical contraindications, patient refusal, lack of appropriate equipment, or if staff have not been trained or are not comfortable with the procedure. Although CSF collection is usually the standard procedure in research studies and good collection rates can be achieved [42], surveillance programs usually involve large numbers of health facilities in geographically dispersed areas and facilities of lower capacity. Although optimal clinical management of any patient with a suspected CNS infection includes a LP unless there are contra-indications [45], especially to exclude treatable conditions like bacterial meningitis, in some parts of Asia CSF is not always examined. CSF collection rates of about 30% have been documented in recent JE surveillance [12,46].

For these reasons, the CWG felt that an AES case with JEV IgM detected in a single sample of either CSF or serum should be considered "laboratory-confirmed" to avoid missing important surveillance information, although CSF is the preferred specimen. *[Category III] *Some cases with JEV IgM in serum and encephalitis due to another cause may occasionally be incorrectly included as clinical JE cases, but this is probably a rare event. Studies show inapparent JEV infection rates in children of about 5% annually [27,47,48], and it is only if JEV IgM is still present after inapparent infection that its detection could result in mistaken attribution of JEV as the cause of the clinical encephalitis illness. In other words, the positive predictive value of a JEV IgM positive result in serum in a patient with encephalitis in a JE-endemic area is likely to be very high. Furthermore, the presence of JEV IgM in serum confirms the patient has been infected and therefore that JEV is circulating in that area. Inclusion of these cases is unlikely to result in an overestimate of JE disease burden if the recognized overall lack of sensitivity of surveillance data is considered [37,49-51].

However, with more widespread introduction of JE vaccination programs, collection of CSF will become increasingly important. After vaccination, JEV IgM may be detectable in serum but is not present in CSF. If a recently-vaccinated person develops AES, their illness may erroneously be attributed to JE if IgM is detected in serum, even though there is no IgM in the CSF. This situation has already been seen in at least one encephalitis outbreak [52].

What tests are appropriate for confirming JE infection?

For routine surveillance purposes an IgM-capture enzyme-linked immunosorbent assay (ELISA) specifically for JEV is considered the standard diagnostic tool [8,26,40]. Other methodologies may be used in reference or research laboratories, but are not recommended for routine surveillance. Usually, attempts to isolate virus or detect viral genome with a nucleic acid amplification test in serum or CSF are unsuccessful, probably because of low viral titres and the rapid development of neutralizing antibodies [53-55]. Occasionally JEV has been isolated, or genome has been detected, in CSF [19,56-60]. Isolates may sometimes be obtained postmortem from brain tissue, and JEV antigens may be detected by immunohistochemistry or immunofluorescence assays in brain tissue or CSF [10,19,29,57,61-63]. Plaque reduction neutralization and haemagglutination inhibition assays can confirm JEV infection but are time-consuming and require acute and convalescent samples to be collected. *[Category II]*(Additional file 2)

What timing should be recommended for collection of samples?

CSF and serum samples should be routinely collected at hospital admission for clinical management purposes, as well as for JE diagnostic testing. However as JEV IgM may not be detectable during early illness, the Standards recommend collection of a convalescent serum sample on the 10^th ^day of illness. In one study involving 60 JE patients, 100% of patients with serum collected on days 9-10 of illness had JEV IgM present but only about 80% with samples collected on days 7-8 had IgM detectable [41]. Another study found 88% (23/26) of patients with samples collected on days 9-12 of illness had detectable JEV IgM compared with 70% (31/44) on days 5-8 and 59% (26/44) on days 1-4 [40]. Patients with JE are typically admitted to hospital 3 to 4 days after illness onset so the 10^th ^day of illness corresponds to day 7 after hospital admission [24,43], and another study, measuring from day of admission, found 100% (19/19) of JE patients had IgM in serum on day 7 after admission compared with 53% on day 1 [39]. Thus collection of a second serum sample on day 10 after illness onset (or 7 days after hospital admission) is recommended. *[Category I] *Although this time point is preferable, if the patient is due to be discharged earlier, or is very unwell and looks unlikely to survive to day 10, then an earlier sample should be collected because any second sample is better than none.

Although collection of CSF and two serum samples is recommended, the availability and timing of laboratory testing may determine the actual testing conducted. For example, if the CSF specimen is tested first and is positive, there is no need to test the sera; if a convalescent serum is positive, the acute serum need not be tested. However in some settings it may be most cost-effective and appropriate to test all samples at the same time.

What factors need to be considered in testing for JEV infection and in interpreting test results?

Antigenic cross-reactivity between flaviviruses is common and an important issue when conducting serological testing for JEV infection. A patient could have a falsely positive JEV IgM result if infected with another flavivirus [41,64-66]. Circulation of dengue virus, a related flavivirus, is common in many JE-endemic areas, and West Nile or Murray Valley encephalitis viruses also co-exist with JEV in some areas [12,67]. It is therefore essential that a representative number of JEV IgM positive samples be tested for other regionally-relevant flaviviruses and/or confirmed by the relevant reference laboratory. *[Category I]*

### Case classification

What case classifications are appropriate based on clinical, epidemiological and laboratory findings?

All cases that meet the AES definition (termed "suspected JE cases") should be included in syndromic AES reporting, regardless of whether an aetiology other than JE is identified. Classification of AES cases into one of four sub-groups--laboratory-confirmed, probable JE, AES-other agent, or AES-unknown--is based on laboratory testing and epidemiological findings (Additional file 3). The rationale for including all cases in AES syndromic reporting prior to assigning the specific classification is that the identification of a particular causal aetiology relies on access to diagnostic tests, and there is marked variation in capacity for laboratory testing for encephalitis aetiologies in individual laboratories and from country to country. Thus a patient that meets the AES case definition and has a laboratory-confirmed diagnosis of cerebral malaria, for example, should be reported as "AES" and classified as "AES-other agent". If a consistent AES definition is not applied, regardless of diagnostic capabilities, variations in reported AES case numbers from site to site could result, unrelated to actual disease burden. Reporting all AES cases also provides evidence that the surveillance is active, even if no JE cases are occurring.

The classification of "probable JE" enables clear categorization of JE cases during an outbreak. The Standards propose that after a seasonal outbreak has been laboratory-confirmed as JE, it may not be necessary to test all remaining outbreak cases (see "Notes", Additional file 2). However, classifying these cases as "probable JE" (i.e., having a geographic and temporal link to a laboratory-confirmed JE case) differentiates them from non-specific "AES-unknown" cases. This is useful to document the extent of an outbreak.

### Recommended types of surveillance

What is the best model for JE surveillance and are different types of surveillance appropriate in different settings?

Nationwide syndromic AES surveillance with laboratory testing of all patients provides the best information on JE disease burden, epidemiology, and the impact of vaccination programmes. However, in some countries, this is logistically complex and would result in a significant financial burden for the public health system. In such settings, sentinel surveillance may be the appropriate strategy. *[Category III] *As most AES cases will present to hospitals, sentinel surveillance can be hospital-based. In conjunction with nationwide syndromic AES surveillance--reporting from all health facilities of clinical AES cases--a selected number of "sentinel" hospitals should conduct case-based surveillance with laboratory testing of cases (Additional file 4). This provides information on the proportion of all AES cases due to JE. If assumptions can be made that the sentinel site populations are representative of larger geographical areas and that surveillance is functioning with reliable completeness and accuracy at these sites, then information on the proportion of AES cases confirmed to be JE can be used for broader extrapolations. Using nationwide AES data, for example, national JE incidence estimates can be approximated. Although this strategy has limitations, the estimates enable monitoring of JE incidence over time. A possible disadvantage of the sentinel surveillance strategy is that if sentinel hospitals are not located in appropriate geographical locations or are insufficient in number, JE disease transmission may be missed. In addition, JE-specific information to monitor immunization programme performance may not be available to provincial- or district-level EPI managers.

### Minimum data elements, analyses and reporting

What data should be collected for analysis and reporting for routine surveillance purposes?

Epidemiological and laboratory data elements recommended for collection to enable understanding of disease burden and monitoring of immunization programs are provided in the Standards. Variables include age, sex, date of onset, place of residence, immunization status, travel history, clinical illness details, laboratory results, and information on outcome. Suggested analyses are also defined and can be viewed in full in the Standards. *[Category III]*

What targets should be used to monitor the quality of JE surveillance?

Targets were defined for completeness and timeliness of reporting (≥ 90% and ≥ 80%, respectively); the percentage of cases with specimens collected, a serum sample at least 10 days after onset, and samples reaching the laboratory in adequate condition (≥ 80% for each); and laboratory results reported in less than one month (≥ 80%). These performance indicators were considered feasible based on experience with the use of similar performance indicators for other vaccine-preventable diseases and on the existing status of surveillance in JE-endemic countries. They are only minimum targets and locally-appropriate higher targets should be defined in countries with strong surveillance systems or as systems improve. *[Category III]*

The Standards also include a performance indicator for minimum annual incidence of AES of at least 2 cases per 100,000 population. This is the expected baseline AES incidence even without JEV transmission (i.e., if at least two non-JE AES cases per 100,000 population are reported annually, it suggests the AES surveillance system is functioning adequately). This figure was derived from a limited literature review on encephalitis incidence in industrialized settings where arboviruses are a less prominent cause of encephalitis, thus reflecting encephalitis rates in the absence of JE. A more extensive literature review has since been undertaken, reviewing studies from both industrialized and tropical settings and prioritizing results from prospective studies as they were stronger methodologically [68]. It suggested that appropriate minimum annual AES incidence targets for children under 15 years of age, adults and all age groups were 10, 2 and 6 cases per 100,000, respectively. Limitations were that a variety of case definitions and methodologies were used in the studies; research definitions are likely narrower than the AES definition used in the Standards; no prospective childhood study in a tropical area was available; and epidemiology of acute encephalitis has changed because of immunization programmes for diseases such as measles and mumps. In future, the target for minimum AES incidence may be revised, particularly if additional data become available. *[Category II]*

### Special aspects

What other special aspects of clinical and laboratory surveillance for AES and JE should be considered?

The "Special Aspects" section of the Standards discusses interpretation of laboratory results in a recently-immunized patient, the importance of comprehensive investigation of AES patients to ensure appropriate clinical management, and the potential value of integrated meningoencephalitis surveillance [2].

Testing a single serum sample for JEV IgM within six months of JE vaccination may not be diagnostic for JE illness, as JEV IgM may be present in serum after vaccination [69-72]. The period IgM may remain detectable has not been accurately determined. Unpublished studies have shown IgM detectable for at least two months after immunization with inactivated vaccine (J. Cardosa, UNIMAS Malaysia, personal communication). A study using live, attenuated SA 14-14-2 vaccine demonstrated IgM in 13% of children (9/68) one month after vaccination [71]. After natural JEV infection, IgM persists for 6 months in about 40% of patients, but the IgM response after vaccination is less vigourous [39,69]. Additional data on IgM persistence after live JE vaccine are being gathered to help clarify this issue. *[Category II] *The Standards list tests that are acceptable to confirm JE in a recently-vaccinated patient, in particular testing of CSF. JEV IgM is not present in CSF after vaccination, so detection of antibody in CSF indicates wild JEV infection.

Careful investigation of patients is important to ensure that treatable causes of CNS infection-including malaria, herpes simplex virus infection and bacterial meningitis-are not missed. Because several important causes of bacterial meningitis have become vaccine-preventable, and as there is a clear overlap between patients who meet the WHO case definitions for "AES" and "bacterial meningitis", the concept of integrated meningoencephalitis surveillance is also discussed in the Standards. This approach is consistent with clinical management of meningoencephalitis cases, and possible benefits include improved case detection, reduction in programmatic duplication, streamlining of logistics, and better use of resources [73].

## Discussion

This paper has documented the process undertaken for developing and refining the WHO JE surveillance standards, described the rationale for the recommendations made, and provided the supporting documentation. It also describes the level of evidence for each topic addressed. This is expected to support credibility of the Standards and improve understanding and acceptance of the recommendations [74].

The CWG focused on developing Standards with feasible recommendations so implementation is possible in Asian countries. Strong surveillance is important for documenting JE disease burden to assist with decision-making on vaccine introduction and for monitoring immunization programmes. It is anticipated this paper will support and facilitate successful implementation of the WHO JE surveillance standards.

## Competing interests

The authors declare that they have no competing interests.

## Authors' contributions

SH drafted the manuscript with considerable input from TS. All authors contributed substantially to the development and refinement of the WHO JE surveillance standards, and to critically reviewing and approving the content of this manuscript. Other members of the JE Core Working Group contributed to development of, and provided feedback to assist in refining, the JE surveillance standards.

## Pre-publication history

The pre-publication history for this paper can be accessed here:

http://www.biomedcentral.com/1471-2334/9/214/prepub

## Supplementary Material

Additional file 1 **Recommended clinical case definition for surveillance**. The recommended clinical case definition for surveillance for cases of acute encephalitis syndrome. Click here for file

Additional file 2 **Laboratory criteria for confirmation of Japanese encephalitis (JE)**. Laboratory criteria for diagnosis of JE in a case of acute encephalitis syndrome. Click here for file

Additional file 3 **Case classification in acute encephalitis syndrome (AES)**. The classification system for AES cases, based on laboratory and epidemiological criteria. Click here for file

Additional file 4 **Recommended types of surveillance**. Recommended models for JE surveillance in different settings. Click here for file
