# Supplementary material for: Evidence and rationale for the World Health Organization recommended standards for Japanese encephalitis surveillance
Source: BMC Infect Dis. 2009 Dec 29;9:214. doi: 10.1186/1471-2334-9-214 (PMC2809064; doi:10.1186/1471-2334-9-214)
Supplement: Additional file 1 — Recommended clinical case definition for surveillance. The recommended clinical case definition for surveillance for cases of acute encephalitis syndrome. [file 1471-2334-9-214-S1.DOC]

**Additional file 1**

**Recommended clinical case definition for surveillance**

Clinically, a case of Acute Encephalitis Syndrome (AES) is defined as a person of any age, at any time of year with the acute onset of fever and at least one of:

a) change in mental status (including symptoms such as confusion, disorientation, coma, or inability to talk);

b) new onset of seizures (excluding simple febrile seizures [1]).

Other early clinical findings may include an increase in irritability, somnolence or abnormal behaviour greater than that seen with usual febrile illness [2].

[1] A simple febrile seizure is defined as a seizure that occurs in a child aged 6 months to less than 6 years old, whose only finding is fever and a single generalized convulsion lasting less than 15 minutes, and who recovers consciousness within 60 minutes of the seizure.

[2] JE virus infection can also sometimes present with a meningitis syndrome or an acute limb paralysis syndrome, which are not covered in these clinical case definitions.
