# Supplementary material for: Evidence and rationale for the World Health Organization recommended standards for Japanese encephalitis surveillance
Source: BMC Infect Dis. 2009 Dec 29;9:214. doi: 10.1186/1471-2334-9-214 (PMC2809064; doi:10.1186/1471-2334-9-214)
Supplement: Additional file 2 — Laboratory criteria for confirmation of Japanese encephalitis (JE). Laboratory criteria for diagnosis of JE in a case of acute encephalitis syndrome. [file 1471-2334-9-214-S2.DOC]

**Additional file 2**

**Laboratory criteria for confirmation of Japanese encephalitis (JE)**

Clinical signs of JE are indistinguishable from other causes of acute encephalitis syndrome (AES). Laboratory confirmation is therefore essential for accurate diagnosis of JE. Detection of IgM antibody by capture ELISA in cerebrospinal fluid (CSF) or serum reaches >95% sensitivity 10 days after onset of first symptoms (see note below).

The recommended method for laboratory confirmation of a JE virus infection is:

1. Presence of JE virus-specific IgM antibody in a single sample of CSF or serum, as detected by an IgM-capture ELISA specifically for JE virus [1].

In addition, any of the following laboratory criteria is confirmatory for JE:

1. Detection of JE virus antigens in brain tissue by immunohistochemistry or immunofluorescence assay; OR
2. Detection of JE virus genome in CSF, serum, plasma, blood [2], or brain tissue by reverse transcriptase polymerase chain reaction (PCR) or an equally sensitive and specific nucleic acid amplification test; OR
3. Isolation of JE virus in CSF, serum, plasma, blood [2] or brain tissue; OR
4. Detection of a four-fold or greater rise in JE virus-specific antibody as measured by haemagglutination inhibition (HI) or plaque reduction neutralization assay (PRNT) in serum collected during the acute and convalescent phases of illness. The two specimens for IgG should be collected at least 14 days apart .These should be performed in parallel with other flaviviruses as indicated in footnote [1].

**Note:**

- CSF is the preferred sample for diagnosis of JE.
- The large majority of JE infections are asymptomatic. Therefore, in areas that are highly endemic for JE, it is possible to have AES due to a cause other than JE virus and have JE virus-specific IgM antibody present in serum. To avoid implicating asymptomatic JE as the cause of other AES illnesses, sterile collection and testing of a CSF sample from all persons with AES is recommended when feasible.
- A serum sample should be obtained at admission. Because it may not yet be positive in a JE-infected person, a second serum sample should be collected at discharge or on the 10th day of illness onset (usually around 7 days after admission) or at the time of death and tested for presence of JE virus-specific IgM.
- It is not necessary to test all specimens in a normal seasonal outbreak of JE after the outbreak has been confirmed by laboratory testing. If the outbreak is not an expected seasonal outbreak, or there are unusual epidemiological features (e.g., age distribution of cases not consistent with pattern of JE infection), testing of CSF is especially important, as an encephalitis outbreak could be due to other etiologies.

[1] Further confirmatory tests (e.g., looking for cross-reactivity with other flaviviruses circulating in the geographical area) should be carried out when: a) There is an ongoing dengue or other flavivirus outbreak; b) when vaccination coverage is very high; c) or in cases in areas not having epidemiological and entomological data supportive of JE transmission.

[2] Detection of virus genome or virus isolation in serum, plasma, or blood is very specific for JE diagnosis; however, it is not sensitive as virus levels are usually undetectable in clinically ill JE cases. Therefore a negative result by these methods should not be used to rule out JE in a suspected case. Similarly detection of virus genome or virus isolation in CSF is usually only found in fatal cases and therefore not very sensitive and should not be used for ruling out a diagnosis of JE.
