# Supplementary material for: Evidence and rationale for the World Health Organization recommended standards for Japanese encephalitis surveillance
Source: BMC Infect Dis. 2009 Dec 29;9:214. doi: 10.1186/1471-2334-9-214 (PMC2809064; doi:10.1186/1471-2334-9-214)
Supplement: Additional file 3 — Case classification in acute encephalitis syndrome (AES). The classification system for AES cases, based on laboratory and epidemiological criteria. [file 1471-2334-9-214-S3.DOC]

**Additional file 3**

**Case classification in acute encephalitis syndrome (AES)**

**AES (Suspected JE) case:** A case that meets the clinical case definition for AES.

AES cases should be classified in one of the following four ways:

- **Laboratory-confirmed JE:** An AES case that has been laboratory-confirmed as JE.
- **Probable JE:** An AES case that occurs in close geographical and temporal relationship to a laboratory-confirmed case of JE, in the context of an outbreak.
- **AES-other agent:** An AES case in which diagnostic testing is performed and an etiologic agent other than JE virus is identified.
- **AES-unknown:** An AES case in which no diagnostic testing is performed, or in which testing was performed but no etiologic agent was identified, or in which the test results were indeterminate.
