# Supplementary material for: Evidence and rationale for the World Health Organization recommended standards for Japanese encephalitis surveillance
Source: BMC Infect Dis. 2009 Dec 29;9:214. doi: 10.1186/1471-2334-9-214 (PMC2809064; doi:10.1186/1471-2334-9-214)
Supplement: Additional file 4 — Recommended types of surveillance. Recommended models for JE surveillance in different settings. [file 1471-2334-9-214-S4.DOC]

**Additional file 4**

**Recommended types of surveillance**

JE surveillance should be conducted year-round. Where feasible, surveillance for and reporting of JE should be performed within the context of integrated disease surveillance and linked synergistically with similar surveillance activities, such as those for AFP or meningitis.

A. In all Asian countries:

Comprehensive syndromic surveillance for AES with aggregate reporting is recommended. In sentinel hospitals, surveillance should be case-based with specimens collected for laboratory confirmation. The number of sentinel hospitals can be gradually increased if feasible logistically.

B. In Asian countries where a high level of JE control has been achieved:

Surveillance should be case-based throughout the country and include laboratory confirmation of all suspected cases.

Regardless of the type of surveillance, reporting should be weekly or monthly and include "zero-reporting" (i.e., no blanks should be left in the reporting forms; a zero should be indicated when there are no cases detected). Outbreak investigations should be initiated if there is a sudden increase in cases or if cases reported are different from historical information in terms of season, geographical area, age group, or case fatality.
